# Pathogenesis, Immunology, and Diagnosis of Latent *Mycobacterium tuberculosis* Infection

**DOI:** 10.1155/2011/814943

**Published:** 2010-12-27

**Authors:** Suhail Ahmad

**Affiliations:** Department of Microbiology, Faculty of Medicine, Kuwait University, P.O. Box 24923, Safat 13110, Kuwait

## Abstract

Phagocytosis of tubercle bacilli by antigen-presenting cells in human lung alveoli initiates a complex infection process by *Mycobacterium tuberculosis* and a potentially protective immune response by the host. *M. tuberculosis* has devoted a large part of its genome towards functions that allow it to successfully establish latent or progressive infection in the majority of infected individuals. The failure of immune-mediated clearance is due to multiple strategies adopted by *M. tuberculosis* that blunt the microbicidal mechanisms of infected immune cells and formation of distinct granulomatous lesions that differ in their ability to support or suppress the persistence of viable *M. tuberculosis*. In this paper, current understanding of various immune processes that lead to the establishment of latent *M. tuberculosis* infection, bacterial spreading, persistence, reactivation, and waning or elimination of latent infection as well as new diagnostic approaches being used for identification of latently infected individuals for possible control of tuberculosis epidemic are described.

## 1. Introduction

Tuberculosis (TB) has afflicted mankind from the time immemorial. Evidence of spinal disease has been found in Egyptian mummies of several thousand years BC and references to TB are found in ancient Babylonian and Chinese writings. Recent molecular genetic studies have shown that *Mycobacterium tuberculosis*, the most common cause of TB in humans worldwide, has a progenitor ~3 million years old [1]. *Mycobacterium tuberculosis* is a member of the *M. tuberculosis* complex (MTBC) which includes six other closely related species: *M. bovis, M. africanum, M. microti, M. pinnipedii, M. caprae,* and *M. canetti*. Although all MTBC members are obligate pathogens and cause TB, they exhibit distinct phenotypic properties and host range. The MTBC members are genetically extremely closely related, the genome of *M. tuberculosis* shows <0.05% difference with *M. bovis,* the latter species primarily infects cattle but can also cause TB in other mammals including humans [2, 3]. 

Tuberculosis is one of the most prevalent infections of human beings and a formidable public health challenge that shows little sign of abating. The disease contributes considerably to illness and death around the world, exacting a heavy toll on the world's most vulnerable citizens. The current TB epidemic is being sustained and fuelled by two important factors: the human immunodeficiency virus (HIV) infection and its association with active TB disease and increasing resistance of* Mycobacterium tuberculosis* strains to the most effective (first-line) anti-TB drugs [4]. Other contributing factors include population expansion, poor case detection and cure rates in impoverished countries, active transmission in overcrowded hospitals, prisons, and other public places, migration of individuals from high-incidence countries due to wars or famine, drug abuse, social decay, and homelessness. Active disease patients with sputum smear-positive pulmonary TB are the main source of infection in a community. Primary infection with *M. tuberculosis* leads to clinical disease in only ~10% of individuals. In the remaining cases, the ensuing immune response arrests further growth of *M. tuberculosis*. However, the pathogen is completely eradicated in only ~10% people, while the immune response in the remaining ~90% individuals only succeeds in containment of infection as some bacilli escape killing by blunting the microbicidal mechanisms of immune cells (such as phagosome-lysosome fusion, antigen presentation by MHC class I, class II, and CD1 molecules, production of nitric oxide, and other reactive nitrogen intermediates) and remain in nonreplicating (dormant or latent) state in old lesions. The process is termed as latent tuberculosis infection (LTBI), and the dormant bacilli retain the ability to resuscitate and to cause active TB if a disruption of immune response (as in HIV infection) occurs. The World Health Organization (WHO) has estimated that one-third of the total world population is latently infected with *M. tuberculosis* and 5%–10% of the infected individuals will develop active TB disease during their life time [4, 5]. However, the risk of developing active disease is 5%–15% every year and lifetime risk is *∼*50% in HIV coinfected individuals [4, 6]. Most of the active disease cases in low TB incidence countries arise from this pool of latently infected individuals.

According to WHO estimates, 9.27 million new active disease cases corresponding to an estimated incidence of 139 per 100,000 population occurred throughout the world in 2007 [4]. Only 5.5 million of 9.27 million cases of TB (new cases and relapse cases) were notified to national tuberculosis programs of various countries, while the rest were based on assessments of effectiveness of surveillance systems. The highest number of TB cases occurred in Asia (55%) followed by Africa (31%). The highest incidence rate (363 per 100,000 population) was recorded for the African region, mainly due to high prevalence of HIV infection. The six most populous countries of Asia (China, India, Indonesia, Pakistan, Bangladesh, and Philippines) accounted for >50% of all TB cases worldwide. An estimated 1.37 million (15%) of incident TB cases in 2007 were coinfected with HIV. Nearly 80% of the HIV-infected TB patients were living in the African region [4]. Globally, 13.7 million total prevalent TB cases were recorded in 2007 corresponding to 206 cases per 100 000 population that resulted in 1.756 million deaths (including 456 000 among TB patients coinfected with HIV) [4]. Nearly 500 000 cases of multidrug-resistant TB (MDR-TB, defined as infection with *M. tuberculosis* strains resistant at least to the two most important first-line drugs, rifampin and isoniazid) occurred in 2007 [4]. By the end of 2008, extensively drug-resistant TB (XDR-TB; defined as MDR-TB strains additionally resistant to a fluoroquinolone and an injectable agent such as kanamycin, amikacin, viomycin, or capreomycin) has been found in 55 countries and territories of the world [4]. While MDR-TB is difficult and expensive to treat, XDR-TB is virtually an untreatable disease in most of the developing countries [7].

Population-based studies have shown that some individuals are more at risk of acquiring infection and developing active disease than others. Active transmission also occurs more frequently in small households and crowded places in countries with a high incidence of TB [8, 9]. Molecular epidemiological studies have shown that there are distinct differences in the disease presentation and population demographics in low TB incidence and high TB incidence countries. In several African and Asian countries, where the transmission of *M. tuberculosis* has been stable or increased in the last few years, the incidence rate is highest among young adults with most cases resulting from recent episodes of infection or reinfection. On the contrary, in low TB incidence countries of Western Europe and North America, a higher proportion of cases occur in older patients or among immigrants from high TB incidence countries [8, 10]. Pulmonary TB accounts for >85% of active TB cases in high TB incidence countries due to higher rates of active transmission, while extrapulmonary TB is also common in low TB incidence countries of the developed world, particularly among HIV-infected individuals and immigrants originating from TB endemic countries [11, 12].

## 2. Transmission of *M. tuberculosis* Infection

**Figure 1:**
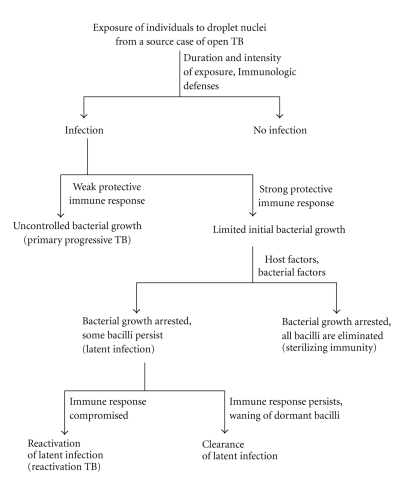
Progression of natural course of events and outcome in an immunocompetent individual following exposure to droplet nuclei containing *M. tuberculosis* expectorated by a source case of sputum smear-positive pulmonary (open) TB.

Tuberculosis is a communicable disease and patients with pulmonary TB are the most important source of infection. Infection is initiated by inhalation of droplet nuclei, which are particles of 1–5 *μ*m in diameter containing *M. tuberculosis*, expectorated by patients with active pulmonary TB (open TB), typically when the patient coughs. The droplet nuclei, due to their small size, can remain suspended in the air for several minutes to hours. The risk of infection (Figure 1) is dependant on several factors such as the infectiousness of the source case, the closeness of contact, the bacillary load inhaled, and the immune status of the potential host [8–10]. The primary route of infection involves the lungs. Inhaled droplet nuclei avoid the defenses of the bronchi due to their small size and penetrate into the terminal alveoli where they are engulfed by phagocytic immune cells (macrophages and dendritic cells). *M. tuberculosis* can also infect nonphagocytic cells in the alveolar space including M cells, alveolar endothelial, and type 1 and type 2 epithelial cells (pneumocytes) [13–19]. In the early phase of infection, *M. tuberculosis,* internalized by phagocytic immune cells, replicates intracellularly, and the bacteria-laden immune cells may cross the alveolar barrier to cause systemic dissemination [14, 15]. The intracellular replication and simultaneous dissemination of the pathogen to the pulmonary lymph nodes and to various other extrapulmonary sites occur prior to the development of the adaptive immune responses. This exemplifies the extraordinary ability of *M. tuberculosis* to establish a protected niche where they can avoid elimination by the immune system and to persist indefinitely [20, 21]. 

In the vast majority of the infected individuals, an effective cell-mediated immune response develops 2–8 weeks after infection that stops further multiplication of the tubercle bacilli (Figure 1). The activated T lymphocytes, macrophages, and other immune cells form granulomas that wall off the growing necrotic tissue limiting further replication and spread of the tubercle bacilli. Most of the *M. tuberculosis* are killed in the caseating granulomas, and disease progression is arrested. However, the pathogen is not completely eradicated in some individuals as *M. tuberculosis* has evolved effective strategies to evade the immune response resulting in survival and persistence of some bacilli in a nonreplicating state in the host (LTBI) [8, 21, 22]. In support of this hypothesis, *M. tuberculosis* has been cultured and presence of* M. tuberculosis* DNA has been demonstrated from lung tissues of individuals who died from other diseases and who did not exhibit any pathological sign of TB disease [23, 24]. Furthermore, a recent report showing transmission of infection from father to son in Denmark in 1961 and reactivation of latent infection in the son more than 30 years later (documented by molecular fingerprinting of their respective *M. tuberculosis* isolates) has indicated that the surviving bacilli may remain dormant for a long time (lasting up to a lifetime) [25]. A subsequent defect in cell-mediated immunity may result in reactivation of dormant bacilli causing active disease many years after the infection (reactivation TB). The current understanding of the mechanisms leading to the establishment of latent infection and the transition back to active growth in reactivation of latent disease is described below.

## 3. Entry Mechanisms of *M. tuberculosis*

Infection with *M. tuberculosis* starts with phagocytosis of the bacilli by phagocytic antigen-presenting cells in the lung including alveolar macrophages and dendritic cells. The recognition of pathogen-associated molecular patterns (PAMP) by specific pathogen recognition receptors (PRRs) is central to the initiation and coordination of the host innate immune response [26].* M. tuberculosis* internalized through different receptors may also have different fate. 

The *M. tuberculosis* or *M. tuberculosis* components are recognized by host receptors that include Toll-like receptors (TLRs), nucleotide-binding oligomerization domain- (NOD-) like receptors (NLRs), and C-type lectins. The C-type lectins include mannose receptor (CD207), the dendritic cell-specific intercellular adhesion molecule grabbing nonintegrin (DC-SIGN) and Dectin-1 [27, 28]. Other potential receptors include complement receptors, scavenger receptors, surfactant protein A receptors (Sp-A), and cholesterol receptors [29]. Some of these receptors (such as TLRs) are expressed on both, immune cells (such as macrophages, dendritic cells, B cells, and specific types of T cells) and nonimmune cells (like fibroblasts and epithelial cells). The interaction of *M. tuberculosis* with TLRs initiates an intracellular signaling cascade that culminates in a proinflammatory response (beneficial to the host); however, the bacterium has also evolved strategies that can trigger signals that dampen or modulate the innate immune response (beneficial to the pathogen). Other membrane bound PRRs (CD207, DC-SIGN, and Dectin-1) contribute to the relay of inflammatory signals while cytosolic PRRs (such as Nod-like receptor) modulate host recognition of the pathogen [27, 28]. 

The TLR engagement, particularly TLR2 and TLR4, with *M. tuberculosis*/*M. tuberculosis* component is an early event in the interaction of the pathogen with host cells and TLR signaling is the main arm of the innate immune response during *M. tuberculosis* infection [27, 28, 30]. The TLR polymorphisms regulate the innate immune response to mycobacterial lipopeptides and clinical susceptibility to pathogens [31]. Typically, signals generated by the interactions of TLRs with ligands on *M. tuberculosis* induce the activation of proinflammatory and antimicrobial innate immune response.

The *M. tuberculosis* cell envelope is composed of a cell wall that is covered with a thick waxy mixture of lipids and polysaccharides and is characterized by a high content of mycolic acids. The most important *M. tuberculosis* cell surface ligands that interact with TLRs and other receptors include the 19 and 27 kDa lipoproteins, 38 kDa glycolipoprotein, the lipomannan (LM) and mannose-capped lipoarabinomannan (ManLAM) [32–34]. Other ligands include LprA and LprG lipoproteins [35, 36] and, perhaps also, surface-exposed mammalian cell entry (Mce) proteins encoded by *mce1* and *mce3* operons [37–39]. The interaction of *M. tuberculosis* ligand(s) with TLRs eventually results in activation of nuclear transcription factor (NF)-*κ*B and production of proinflammatory cytokines such as tumor necrosis factor (TNF)-*α*, interleukin (IL)-1, IL-12, chemokines, and nitric oxide through either myeloid differentiation primary response protein 88 (MyD88)-dependant or MyD88-independent pathway [27, 34, 40, 41]. 

Restricting TLR-induced proinflammatory signals is essential to avoid the risk of producing excessive inflammation that could damage host tissues. A family of receptor tyrosine kinases termed Tyro3/Axl/Mer (TAM) provide a negative feedback mechanism to both TLR-mediated and cytokine-driven proinflammatory immune responses [42]. This property has been exploited by *M. tuberculosis* to its advantage. The 19 kDa lipoprotein of *M. tuberculosis* is an agonist of the TLR2 and modulates the innate immunity and antigen presenting cell function [32]. Studies have shown that prolonged TLR2 signaling by lipoproteins of *M. tuberculosis* inhibits major histocompatibility complex (MHC)-II expression and processing of antigens by macrophages [43, 44]. Thus, a subset of infected macrophages with decreased antigen-presenting cell function may be unable to present *M. tuberculosis* antigens to CD4^+^ T cells resulting in insufficient activation of effector T cells leading to evasion of immune surveillance and creation of niches where *M. tuberculosis* survives and persists [27, 32].

The mannose receptors interact with ManLAM present in the cell envelop of *M. tuberculosis*. The phagocytosis of tubercle bacilli by macrophages through mannose receptor is associated with an anti-inflammatory response as ManLAM inhibits mannose receptor-dependant IL-12 production. This inhibition of macrophage response to *M. tuberculosis* promotes infection and subsequent survival of *M. tuberculosis* in macrophages. The ManLAM exerts its effects on phagolysosome maturation by limiting phagosome-lysosome fusion [45, 46].

## 4. Immune Response of the Host to *M. tuberculosis*

The alveolar macrophages, after entry of *M. tuberculosis*, produce inflammatory cytokines and chemokines that serve as a signal for infection. The monocytes, neutrophils, and lymphocytes migrate to the focal site of infection, but they are unable to kill the bacteria efficiently. During this time, the bacilli resist the bactericidal mechanisms of the macrophage (phagolysosome) by preventing phagosome-lysosome fusion, multiply in the phagosome, and cause macrophage necrosis [47]. The released bacilli multiply extracellularly, are phagocytosed by another macrophage that also fails to control the growth of *M. tuberculosis,* and likewise are destroyed. In the meantime, dendritic cells with engulfed bacilli mature, migrate to the regional lymph node, and prime T cells (both CD4^+^ and CD8^+^) against mycobacterial antigens [48]. The specific immune response produces primed T cells which migrate back to the focus of infection, guided by the chemokines produced by the infected cells. The accumulation of macrophages, T cells, and other host cells (dendritic cells, fibroblasts, endothelial cells, and stromal cells) leads to the formation of granuloma at the site of infection [49].

The granuloma formation walls off tubercle bacilli from the rest of the lung tissue, limits bacterial spread, and provides microenvironment for interactions among macrophages and other cells of the immune system and the cytokines produced by these cells. The CD4^+^ T cells producing interferon-*γ* (IFN-*γ*) recognize infected macrophages presenting antigens from *M. tuberculosis* and kill them [50]. The infection progression is halted; however, some resistant bacilli capable of surviving under the stressful conditions generated by the host escape killing and enter a state of dormancy and persist by avoiding elimination by the immune system [22, 51, 52]. Recent studies have shown that differences exist in the immunological response mounted by different individuals that lead to the formation of physiologically distinct granulomatous lesions in individuals exposed to *M. tuberculosis*. Some of these lesions suppress (sterilizing immunity) while others promote the persistence of viable *M. tuberculosis* in the microenvironment [53]. Low-dose infection of cynomolgus macaques that reproduce the clinical characteristics of human latent TB leads to the formation of at least two types of tuberculous granuloma [54, 55]. Histopathological studies have shown that the classic caseous granuloma are composed of epithelial macrophages, neutrophils, and other immune cells surrounded by fibroblasts. The central caseous necrotic region in this type of granuloma consists of dead macrophages/other cells and is hypoxic with *M. tuberculosis* residing inside macrophages in the hypoxic center [55, 56]. The other kind of granulomas seen in latent tuberculosis in both humans and cynomolgus macaques are fibrotic lesions, composed almost exclusively of fibroblasts that contain very few macrophages [55]. However, it is not known at present whether *M. tuberculosis* is located inside macrophages or in the fibrotic area in these lesions.

The microenvironment of the granuloma (hypoxia, low pH, presence of nitric oxide and carbon monoxide, etc.) increases the expression of several *M. tuberculosis* genes involved in dormancy induction [57–60]. Recent findings of formation of spore-like structures in *M. bovis* BCG, *M. marinum,* and *M. smegmatis* in response to prolonged stationary phase or nutrient starvation suggest that sporulation may be a general mechanism for mycobacterial dormancy [60–62]. The dormant bacilli can inhabit the granuloma during the lifetime of the host, but are able to resuscitate (or germinate) in the event of local immunodepression. The latent infection in a person without overt signs of the disease is indicated by the delayed-type hypersensitivity (DTH) response to purified protein derivative (PPD) prepared from culture filtrates of *M. tuberculosis* (tuberculin skin test) [8].

## 5. Specific Roles of Immune Cells and Cytokines in *M. tuberculosis* Infection

Studies in animal models and in humans have demonstrated that a wide range of immune components are involved in an effective immune response against *M. tuberculosis*. These include, beside macrophages and dendritic cells, *αβ*-T cells (both CD4^+^ and CD8^+^), CD1 restricted T cells, *γδ*-T cells, and cytotoxic T cells, as well as the cytokines produced by these immune cells [22, 63, 64]. The most important among these are CD4^+^ T cells and the cytokine IFN-*γ*. Although CD4^+^ T cells along with CD8^+^ T cells and the natural killer (NK) cells are the major producers of IFN-*γ*, studies carried out in CD4^+^ deficient mice have shown that it is the early production of IFN-*γ* by CD4^+^ T cells and subsequent activation of macrophages that determine the outcome of infection [65, 66]. The CD4^+^ T cells also play other roles in the defense against infection that is independent of IFN-*γ* production. Depletion of CD4^+^ T cells was associated with the reactivation of infection in a chronically infected mice and resulted in increasing pathological features and death, even though IFN-*γ* levels were still high due to a strong response from CD8^+^ T cells and normal levels of inducible nitric oxide synthase (iNOS) [67]. 

The CD4^+^ T cells carry out several functions that are important to control infection in the granuloma. These include apoptosis of infected macrophages through Fas/Fas ligand interaction, production of other cytokines (such as IL-2 and TNF-*α*), induction of other immune cells (macrophages or dendritic cells) to produce other immunoregulatory cytokines such as IL-10, IL-12, and IL-15, and activation of macrophages through direct contact via CD40 ligand [63, 66, 68, 69]. The CD4^+^ T cells also appear to be critical for the cytotoxic function of CD8^+^ T cells that is mediated by IL-15 [66, 70]. It has also been shown that CD4^+^ T cells can control the intracellular growth of *M. tuberculosis* by a nitric oxide-dependent mechanism that is independent of IFN-*γ* production [66, 71]. Thus, CD4^+^ T cells, in addition to early production of IFN-*γ* appear to have several other secondary functions that are critical in the control of *M. tuberculosis* infection. 

The CD8^+^ T-cells, in addition to producing IFN-*γ* and other cytokines, may also be cytotoxic for *M. tuberculosis*-infected macrophages, and thus play an important role in providing immunity to TB. The CD8^+^ T-cells can directly kill *M. tuberculosis* via granulysin, and facilitate the control of both the acute as well as chronic infection [66, 72]. The abundant presence of *M. tuberculosis*-specific CD8^+^ T cells in latently infected individuals shows that the CD8^+^ T cells also have a role in the control of latent infection. This is also supported by reactivation of latent infection following depletion of CD8^+^ T cells in the Cornell model of latent TB [73]. 

Studies in primate models of TB have shown that unconventional T cells such as CD1 restricted T cells, and *γδ*-T cells also contribute to the protection against TB [64, 74, 75]. The CD1 restricted T cells recognize the glycolipids such as LAM that are abundant in the mycobacterial cell wall while *γδ*-T cells recognize small metabolites containing phosphate (phospholigands) [74]. Although it is well established that mycobacterial antigens in the phagosome of macrophages or dendritic cells are picked up by the MHC class II molecules and presented to CD4^+^ T cells, studies have shown that the phagosomal membrane is also equipped with the MHC class I processing machinery [76, 77]. Also, CD1 proteins have the capability to present lipid antigens and lipopeptides to T cells, and thus play important roles in the immune response against lipid-rich *M. tuberculosis* [75, 78, 79]. Further, the vesicles formed due to apoptosis of *M. tuberculosis*-infected macrophages and containing mycobacterial antigens such as ManLAM, lipoproteins, and so forth are taken up by dendritic cells and presented to the T cells through the MHC class I and CD1 molecules [75, 78, 80].

The IFN-*γ* is the key cytokine for a protective immune response against *M. tuberculosis*. Humans and mice defective in IFN-*γ* or IFN-*γ* receptor genes are more susceptible to *M. tuberculosis* infection [63, 66, 81]. The IFN-*γ*, produced mainly by CD4^+^, CD8^+^ T cells, and the NK cells, synergizes with TNF-*α* and activates macrophages to kill intracellular bacilli. The IFN-*γ* also augments antigen presentation, leading to recruitment of CD4^+^ T-cells and/or cytotoxic CD8^+^ T-cells, which participate in mycobacterial killing and also prevents exhaustion of memory T cells [63, 82]. Furthermore, IFN-*γ* induces the transcription of more than 200 genes in macrophages including the upregulation of MHC class II expression and the production of antimicrobial effectors such as oxygen radicals and nitric oxide. A major effector mechanism responsible for the antimicrobial activity of IFN-*γ* in association with TNF-*α* is the induction of the production of nitric oxide and other reactive nitrogen intermediates (RNI) by macrophages via iNOS [63, 66, 83]. However, some *M. tuberculosis* factor(s), such as the 19-kDa lipoprotein, have the potential to attenuate the response of macrophages to IFN-*γ* by blocking the transcription of a subset of IFN-*γ*-responsive genes (Table 1) [44, 84, 85]. 

TNF-*α*, produced by macrophages, dendritic cells, and T-cells, is another cytokine that has a major protective role against* M. tuberculosis *infection both in mice and humans [104, 105]. Paradoxically, TNF-*α* also contributes significantly to the development of immunopathology associated with TB [52]. Mice deficient in TNF-*α* or TNF-*α* receptors are more susceptible to mycobacterial infections [104]. This cytokine is involved in both immune and immunomodulatory responses and acts in synergy with IFN-*γ* to enhance the expression of iNOS and the antimycobacterial activity of macrophages [63, 83]. TNF-*α* also initiates cell migration and formation of microbicidal granulomas while disruption of TNF-*α* responses leads to overgrowth of the mycobacterial pathogens [63, 66]. The TNF-*α* produced by the infected macrophages induces the expression of chemokines, such as IL-8, MCP-1, and RANTES which provide signals for migration of immune cells to the sites of *M. tuberculosis* infection [106]. Both T cell- and macrophage-derived TNF-*α* are required for sufficient and long-term protection against *M. tuberculosis* infection [107]. The phenolic glycolipid, a virulence factor in the cell wall of a hypervirulent strain of *M. tuberculosis* (W-Beijing family) inhibits the release of pro-inflammatory cytokines TNF-*α*, IL-6 and IL-12 by macrophages [108].

The importance of IL-12 is also evident from increased susceptibility of mice and humans deficient in IL-12 responses to mycobacterial infections [109]. Individuals with defects in the production of IL-12 or its receptor are highly susceptible to active TB disease [110]. The T-cell-derived cytokines, IFN-*γ* and TNF-*α*, are produced abundantly by activated CD4^+^ T cells under the influence of IL-12, and the role of IFN-*γ* and TNF-*α* in activating and augmenting the microbicidal effector functions of phagocytic cells during a protective immune response against *M. tuberculosis* infection is well established [63, 66, 83].

## 6. Antigen Presentation Pathways and Their Modulation by *M. tuberculosis* Components

The tubercle bacilli reside in the phagosome soon after their entry inside alveolar macrophages and dendritic cells. The priming of CD4^+^ T cells for a protective immune response requires the presentation of *M. tuberculosis* antigens through MHC class II pathway. The phagosomal membrane is also equipped with the MHC class I processing machinery [74, 77]. Further, mycobacterial glycolipids, lipids, and other phospholigands may also be presented to the T cells [78, 80]. Some *M. tuberculosis* factors particularly those associated with the cell wall modulate antigen-processing pathways by MHC class I, MHC class II, and CD1 molecules [28, 63, 111]. The ManLAM, trehalose 6,6′-dimycolate (TDM, also known as cord factor), and the 19 kDa lipoprotein downregulate IFN-*γ*-inducible genes including those involved in antigen presentation by MHC class II machinery (Table 1) [32, 86, 94, 111, 112]. Other mechanisms that modulate antigen presentation include antigen processing and binding of peptides to MHC class II molecules [32, 87]. The 19 kDa lipoprotein also inhibits MHC class I antigen processing via Toll-like receptor signaling [88]. It is probable that continuous attenuation of antigen presentation through multiple mechanisms is advantageous for slowly growing pathogens like *M. tuberculosis* [111, 112]. Inhibition of antigen presentation by these mechanisms results in persistence of *M. tuberculosis* inside macrophages [112].

## 7. Dampening of Other Macrophage Functions by *M. tuberculosis* Components

The two major antimycobacterial mechanisms of macrophages include the generation of nitric oxide and other RNI which exert toxic effects on the bacilli and fusion of the phagosomes containing mycobacteria with lysosomes that is bactericidal [83, 92, 113]. The T cell-derived cytokines, mainly IFN-*γ* and TNF-*α*, activate macrophages, which then generate nitric oxide and other RNI by iNOS and are mycobactericidal [83]. Direct demonstration of the presence of nitrotyrosine, an RNI derived from tyrosine and peroxynitrite in the lungs of infected mice, has shown that RNI are formed in tuberculous granuloma. Furthermore, inhibition of iNOS activity or disruption of iNOS gene, required for the production of RNI, not only abolished the protective effect of RNI but also led to reactivation of latent infection in mice [83]. Although these studies point towards an essential role for iNOS in the control of both acute and chronic persistent infection, the RNI generated through these mechanisms is not sufficient to eliminate the bacterium completely.

The protective role of RNI in human TB has also been suggested [92, 114]. Studies have shown that *M. tuberculosis* has evolved several strategies to evade the RNI toxicity. It has been shown that iNOS, a cytoplasmic protein, may be recruited to the phagosomes and this recruitment may be inhibited by *M. tuberculosis* [115]. The *M. tuberculosis* gene, alkyl hydroperoxide reductase subunit C (*ahpC*), detoxifies, in conjunction with some other proteins, the highly reactive peroxynitrite anion (formed by the reaction of nitric oxide with superoxide) [116]. Another potential mechanism for blunting the toxic effects of RNI is the presence of two haemoglobin-like proteins encoded by *glbN* and *glbO* in *M. tuberculosis*. The *glbN* knockout mutant of *M. bovis* BCG was highly attenuated, and its growth, *in vitro*, was also inhibited by nitric oxide under aerobic conditions [117]. Microarray analyses have shown that more than 30 *M. tuberculosis* genes are induced by RNI and hypoxia [59, 118]. Furthermore, hypoxia and inhibition of respiration by nitric oxide induce a dormancy program in *M. tuberculosis* that leads to increased survival and persistence of the pathogen in immune cells [59, 119].

## 8. Phagolysosome Maturation and Its Inhibition by *M. tuberculosis* Components

The phagocytosis of *M. tuberculosis* by macrophages is followed by the maturation of phagosomes containing the pathogen through a series of fusion and fission events with several endocytic vesicles that culminate in a phagolysosome [120, 121]. The fission-fusion events remodel the phagosomal membrane, and the recruitment of vacuolar-proton transporting ATPase (vH^+^-ATPase) lowers the internal pH that allows lysosome-derived acid hydrolases to function efficiently for their microbicidal effect [122, 123]. Furthermore, phagosome maturation is dependant on Ca^+2^ signaling cascade that begins with phosphorylation of sphingosine to sphingosine 1-phosphate by sphingosine kinase resulting in elevation of cytosolic [Ca^+2^] inside macrophages due to release of Ca^+2^ from intracellular stores in the endoplasmic reticulum and continues through Ca^+2^-calmodulin complex-dependant activation of protein kinase II and phosphatidyl inositol 3-kinase (PI-3K). The cascade culminates in phosphorylation of phosphatidyl inositol to phosphatidyl inositol 3-phosphate (PI-3P) by PI-3K in the phagosome membrane and maturation of phagosome to an acidic bactericidal compartment (phagolysosome) after binding of early endosomal antigen-1 (EEA-1) to PI-3P [89, 124–126].

*M. tuberculosis* has also evolved several strategies to avoid the destruction by lysosomal enzymes by disrupting the maturation of bacilli-containing phagosomes into phagolysosmes [46, 90, 92, 127, 128]. Exclusion of vH^+^-ATPase during maturation of phagosomes contributes to the acidification defect that prevents the fusion of phagosomes with lysosomes [122]. Similarly, modulation of Ca^+2^ signaling cascade, such as SapM-mediated hydrolysis and inactivation of PI-3P, inhibits phagosome maturation leading to enhanced intracellular survival of *M. tuberculosis* (Table 1) [101, 126–128].

Other *M. tuberculosis* components such as ManLAM and TDM (Table 1) also affect phagosome maturation by interfering with the tethering and fusion machinery of vesicular transport in mammalian cells and promote persistence of the bacterium inside macrophages [46, 89, 90, 93, 101]. The targets include the soluble N-ethylmaleimide-sensitive factor-attachment protein receptors (SNAREs), the tethering proteins (such as EEA-1), and the Rab family of GTPases [89, 120, 126, 128]. Some of the membrane trafficking processes affected by mycobacterial factors are also affected by HIV during viral budding and this overlap partially contributes towards the synergism observed between AIDS and active TB [129]. Another mechanism by which mycobacteria interfere with phagosome maturation is by retention of the host tryptophan aspartate rich coat protein (TACO) (homolog of coronin-1) on the cytoplasmic side of their phagosomes that likely inhibits the normal process of phagosome-lysosome fusion [99]. The serine/threonine protein kinase G encoded by *pknG* of *M. tuberculosis* (Table 1) is implicated as the potential effector of the inhibition of phagosome-lysosome fusion [100, 111]. 

Another component of the antimicrobial repertoire of macrophages includes lysosomal killing of *M. tuberculosis* mediated by ubiquitin-derived peptides [130]. The ubiquitination destroys tubercle bacilli by autophagy as a ubiquitin-derived peptide impairs the membrane integrity of *M. tuberculosis* that allows nitric oxide to kill more efficiently. On the contrary, decreased outer membrane permeability protects *M. tuberculosis* from killing by ubiquitin-derived peptides [131].

## 9. Apoptosis of Infected Macrophages and Its Inhibition by *M. tuberculosis* Components

The apoptosis of infected macrophages participates in host defense against infection as apoptotic vesicles containing mycobacterial antigens are taken up by dendritic cells for CD8^+^ T cell activation by phagosome-enclosed antigens [79, 80]. The CD8^+^ T cells activated by apoptotic vesicles from *M. tuberculosis*-infected cells produce IFN-*γ*, which causes uninfected macrophages to produce RNI to effectively kill intracellular *M. tuberculosis*. Several *M. tuberculosis*-derived factors are capable of modulating (activating as well as inhibiting) the apoptosis of infected macrophages through differential expression of proapoptotic and antiapoptotic genes [90]. The mycobacterial components modulating apoptosis of macrophages usually target the caspase cascade or the one involving TLRs. The *M. tuberculosis* components that inhibit apoptosis include cell wall components, ManLAM, virulence-related secretion system encoded by *secA2* that transports superoxide dismutase (encoded by *sodA*) to control reactive oxygen intermediates, and NADH dehydrogenase (encoded by *nuoG*) (Table 1) [91, 102, 103]. Two secretory proteins of *M. tuberculosis* encoded by Rv3654c and Rv3655c that inhibit apoptosis of macrophages have also been identified recently [132]. By inhibiting apoptosis of macrophages, *M. tuberculosis* avoids host defenses and escapes from infected cells by causing necrotic cell death [133].

## 10. Escape of *M. tuberculosis* from Phagosome/Phagolysosome

Although it has been known for quite some time that *M. tuberculosis* survives in the phagosome by blocking (or slowing down) its maturation into phagolysosome and persists, one of the mechanism by which it escapes from phagosome/phagolysosome to infect other macrophages and other immune/alveolar cells has been elucidated recently. Initial subtractive hybridization-based studies identified a genomic region, termed region of difference 1 (RD1), that was present in all virulent *M. tuberculosis* and *M. bovis* strains but was absent in the vaccine strain *M. bovis* BCG [2, 134, 135]. Subsequently, it was shown that RD1 is crucial for the virulence of *M. tuberculosis* as it encoded proteins that formed a novel protein secretion system (ESX-1). ESX-1 (type VII secretion system) is involved in the export of several *M. tuberculosis* proteins including two potent T cell antigens encoded by RD1 itself, the 6-kDa early secreted antigenic target (ESAT-6) (encoded by *esxA*) and 10-kDa culture filtrate protein (CFP-10) (encoded by *esxB*) that lack signal sequences for their export [2, 134, 136–140]. 

The importance of ESX-1 secreted proteins in virulence of *M. tuberculosis* has been shown by deletion of RD1 or disruption of ESAT-6 from *M. tuberculosis* genome that resulted in reduced virulence (spreading) both, in cultured macrophages and in mice [140, 141]. Furthermore, the introduction of RD1 genes in *M. bovis* BCG resulted in altered colonial morphology, increased virulence in severely combined immune deficient mice including the formation of granuloma, and longer persistence in immunocompetent mice [142]. In *M. tuberculosis*, ESAT-6 complexes with CFP-10 in 1 : 1 ratio before its export outside the cell but can dissociate from its partner (CFP-10) at lower pH. Individually, ESAT-6, but not CFP-10, can cause disruption of artificial membranes as well as cytolysis [143–145]. ESAT-6 alone has also been shown to associate strongly with liposomes containing dimyristoylphosphatidylcholine and cholesterol (constituents of mammalian cell membranes) and causing destabilization and lysis of liposomes [95, 96, 144]. 

The studies carried out by de Jonge et al. [96] have shown that ESAT-6:CFP-10 complex secreted by live *M. tuberculosis* inside phagosome splits apart when tubercle bacilli are stressed following acidification of phagosome, and ESAT-6 inserts itself into lipid bilayer, causing lysis and escape of *M. tuberculosis* from phagosome. Further studies have shown that ESAT-6 also induces apoptosis of macrophages via the extrinsic (caspase-dependent) pathway by formation of pores in cell membrane [146] and contributes (or helps) in the translocation of *M. tuberculosis* from the phagolysosomes to the cytoplasm in myeloid cells [97]. More recently, ESAT-6 has also been shown to cause cytolysis of type 1 and type 2 alveolar epithelial cells. This ESAT-6-mediated cytolysis was shown to help in the dissemination of *M. tuberculosis* through alveolar wall [19]. The ESAT-6 homolog from *Mycobacterium marinum*, the bacterium that causes tuberculous granuloma in zebrafish, has also been demonstrated to cause lysis of red blood cells and macrophages by forming pores in their membranes [147, 148]. The presence of a capsular layer containing high amounts of proteins that are secreted via the ESX-1 secretion system including ESAT-6 has also been demonstrated in pathogenic mycobacteria recently [98]. Furthermore, ESX-1-associated proteins in the capsular layer enhanced the interaction of mycobacteria with macrophages and also dampened proinflammatory cytokine response of macrophage. These studies have established the role of ESX-1 secretion system and ESAT-6 protein of *M. tuberculosis* in facilitating macrophage infection and subsequent bacterial escape to infect other nearby cells (Table 1).

## 11. Persistence and Reactivation of Latent TB Infection

The hallmark of *M. tuberculosis* infection in humans is the inability of an otherwise effective immune response to completely eliminate the pathogen. The tubercle bacilli have evolved multiple strategies to manipulate infected host cells in order to evade or modify the ensuing immune response so as to avoid elimination and thus persist in the host. As described above, several *M. tuberculosis* factors, ManLAM and 19-kDa lipoprotein notable among them, have been identified that modulate antigen presentation pathways and either blunt the microbicidal functions of macrophages and other immune cells (such as RNI) or prevent their maturation (phagolysosome) (Table 1). 

Two experimental strategies have been employed to identify other *M. tuberculosis* factors, which promote persistence of the pathogen in immune cells including macrophages. One approach involves cloning of *M. tuberculosis* genes in nonpathogenic mycobacteria and studying their increased survival in macrophages or other mammalian cells while the other approach uses knockout mutants of *M. tuberculosis* for selected genes for persistence of the pathogen in macrophages and other immune cells. Several additional *M. tuberculosis* factors promoting persistence or increased survival in mammalian cells have been identified. Some of these factors include phospholipases encoded by *plcA, plcB, plcC, *and* plcD* [149], the two *PhoP* and *PhoQ* regulatory proteins [21], phosphate-binding proteins PstS1 and PstS2 [150], and proteins encoded by *mce *operons [151]. Thus, *M. tuberculosis* has devoted a large part of its genome towards functions that promote its intracellular survival in mammalian cells including macrophages. 

Reactivation of latent infection requires latent *M. tuberculosis* cells to exit dormancy. Several factors can trigger the development of active disease from the reactivation of remote infection, and this typically involve the weakening of the immune system. HIV infection is the most important single risk factor for progression to active disease in adults as it causes depletion of CD4^+^ T cells and functional abnormalities of CD4^+^ and CD8^+^ T-cells which play an important role in providing protection against active TB disease [4, 6]. Likewise, *M. tuberculosis* infection accelerates the progression of asymptomatic HIV infection to acquired immunodeficiency syndrome (AIDS) and eventually to death. This is recognized in the current AIDS case definition as pulmonary or extrapulmonary TB in HIV-infected patient is sufficient for the diagnosis of AIDS. Old age, malnutrition, and medical conditions that compromise the immune system such as poorly controlled diabetes mellitus, renal failure, and therapy with immunosuppressive drugs are other factors that lead to immunodepression and reactivation of a dormant infection [6, 8, 152, 153]. The reactivation TB can occur in any organ system in which the tubercle bacilli were seeded during the primary infection; however, in immunocompetent individuals, the reactivation usually occurs in the upper lobes, where higher oxygen pressure supports good bacillary growth. The lytic transglycosylases known as resuscitation promoting factors (RPFs) and an endopeptidase (RipA) of *M. tuberculosis* have recently been recognized as vital components for revival from latency [154–156].

## 12. Current Dynamic Model of Latent Infection

The LTBI has traditionally been defined as infection with *M. tuberculosis* in foci within granuloma that remain in nonreplicating state but retain their ability to come out of latency and cause active TB if and when a disruption of the immune response occurs [57]. However, recent experimental data supports a dynamic model of LTBI where endogenous reactivation as well as damage response occurs constantly in immunocompetent individuals [157]. The model suggests that during infection, *M. tuberculosis* grows well inside phagosomes; however, some bacilli released from necrotic macrophages in extracellular milieu in developing granulomas stop replicating. The arrest of bacterial growth occurs even before an effective immune response has fully been developed due to hypoxic and acidic environment (conditions that mimic stationary bacterial cultures) in the extracellular milieu and release of bactericidal enzymes from dead macrophages and neutrophils. The actively growing bacillary population is eventually killed due to the development of an effective immune response; however, nonreplicating bacilli resist killing and survive [158, 159].

The model further suggests that some macrophages (foamy macrophages) also emerge during the chronic inflammatory process, as they have phagocytosed the cellular debris rich in fatty acids and cholesterol derived from cellular membranes [160, 161]. The foamy macrophages also phagocytose extracellular nonreplicating lipid-rich *M. tuberculosis*; however, the bacilli do not grow in the intracellular environment of activated macrophages but are also not killed due to the nonreplicating state of the bacilli [162]. The nonreplicating bacilli-laden foamy macrophages drain from lung granuloma towards the bronchial tree and return to a different region of lung parenchyma due to aerosols generated by inspired air and begin the infection process at this new location once again [157, 160, 161, 163]. In this dynamic process, reinfection in the upper lobe may have the chance to cause cavitary lesion. This is aided by higher oxygen pressure in the upper lobes that can support rapid extracellular bacillary growth resulting in bacillary concentration that can not be controlled by the optimum immune response by the host. The subsequent much stronger inflammatory response leads to tissue destruction, liquefaction, and extracellular bacillary growth which amplifies the response further and causes cavitation [157, 158].

The dynamic infection process leading to active disease in the upper lobes has some parallels with immune reconstitution inflammatory syndrome and the active TB disease that occurs in HIV-infected patients. The presence of bacilli is tolerated by the HIV-infected patients with low CD4^+^ cell counts as the host is unable to mount an inflammatory response needed to control their growth. However, the sudden increase in CD4^+^ T cells in AIDS patients receiving highly active antiretroviral treatment causes an aggressive granulamatous response and active TB disease [164, 165]. The possibility of slow clearance of latent infection proposed by the dynamic infection model has also been supported by a recent study from Norway comprising a population of individuals exposed to a minimal risk of active transmission of infection. Cohort analysis of data from National Tuberculosis Registry to calculate rates and changes in rates of active TB disease, among patients previously exposed to *M. tuberculosis*, has shown that the rate of reactivated TB has progressively decreased over time [166]. The study further suggested that the number of individuals with latent infection could be reduced in half in approximately 9 years in populations in which new infections are effectively prevented. The dynamic infection model also explains how therapy for a relatively short time (9 months) with a single drug (isoniazid), active only against actively dividing bacilli [167], is highly effective for a latent infection that can possibly remain dormant for the entire lifetime of the host. As isoniazid will prevent episodes of reinfection by bacilli resuscitated from dormancy, slow drainage and destruction of nonreplicating bacilli in the stomach will eventually lead to clearance of latent infection [157, 166].

## 13. Diagnosis of Latent *M. tuberculosis* Infection

The persons infected with *M. tuberculosis* may be identified by tuberculin skin test six to eight weeks after exposure to the bacilli. The test is based on a delayed-type hypersensitivity (DTH) response to a complex cocktail of *M. tuberculosis* antigens, known as purified protein derivative (PPD). The induration of more than 5 mm, recorded 48 to 72 hours after injection of PPD, is considered as positive. Surveys conducted with PPD skin test suggest that nearly a third of the world's and half of Asia's population is infected with *M. tuberculosis* [5]. Skin test reaction over 20 mm is usually due to active disease; however, a negative skin test in an active TB patient may also result from anergy or incorrect administration of the test. The tuberculin skin test lacks sensitivity and specificity as it can not differentiate between infection with *M. tuberculosis* and sensitization with other environmental mycobacteria [5, 8]. Also, BCG vaccination may cause false-positive reactions, but these generally last only a few years after vaccination and are in the moderate range (5 to 10 mm).

More sensitive and specific tests such as cell-mediated immunity-based interferon-gamma (IFN-*γ*) release assays (IGRAs) have also been developed that detect T cell responses after stimulation by two *M. tuberculosis*-specific antigens, early secreted antigenic target-6 (ESAT-6) and culture filtrate protein-10 (CFP-10) [168–173]. The IGRAs have excellent specificity as the antigens (ESAT-6 and CFP-10) used in these assays are encoded by genes deleted in the vaccine strain *M. bovis* BCG and majority of environmental mycobacteria of clinical relevance [134, 135, 170]. Another variation of conventional IGRAs has also been developed by using flow cytometry [174]. Although flow cytometric approach has an advantage over conventional IGRAs as a smaller blood volume (<1 ml) is required for testing, the assay has limited utility in much of the developing world due to the requirement of technical expertise and the high cost of flow cytometers. The detection of significant levels of antibodies to some *M. tuberculosis*-specific proteins has also been noted in latently infected individuals as well as in patients with active TB disease but not in healthy subjects [175–178]. However, antibody-based tests have not been used so far for the detection of LTBI.

Two commercial IGRAs, whole blood, ELISA-based QuantiFERON (QFN)-TB Gold assay (Cellestis Ltd., Carnegie, Australia) and peripheral blood mononuclear cell (PBMC) and enzyme-linked immunospot (ELISPOT) technology-based T SPOT-TB (Oxford Immunotec, Oxford, UK) test have also been developed and approved by Food and Drug Administration (FDA) for detecting LTBI. The tests were initially based on stimulation of T lymphocytes with ESAT-6 and CFP-10 proteins and measurement of IFN-*γ* production (QFN-TB Gold) or detection of T-cells themselves (T SPOT-TB). These tests have undergone further improvement. The newer version of the QFN-TB Gold is called QuantiFERON-TB-Gold-In-Tube (QFT-G-IT) (Cellestis Ltd., Carnegie, Australia) that uses ESAT-6 and CFP-10 and TB7.7 (corresponding to Rv2654 [2]) peptides as antigens. The newer version of T-Spot-TB also uses peptides of ESAT-6 and CFP-10 instead of whole ESAT-6 and CFP-10 proteins as antigens (Oxford Immunotec, Oxford, UK). The performance of both QFT-G-IT and T-Spot-TB tests have recently been evaluated extensively, and several systematic reviews are available for a more detailed description [173, 179–182]. Although IGRAs can not distinguish between LTBI and active TB disease in immunocompetent adults [173], in high-risk individuals with immunosuppressive conditions and children, IGRAs may help in the diagnosis of active disease as adjunctive diagnostic tests, particularly if specimens from the suspected site of infection (such as bronchoalveolar lavage, cerebrospinal fluid) rather than blood is used [183, 184]. While the results of IGRAs exhibit better correlation with surrogate measures of exposure to *M. tuberculosis* in low TB incidence countries; however, their performance is suboptimal in countries with a high TB incidence [173, 180–182, 185]. 

In low TB incidence countries of North America and Western Europe, the majority of active TB disease cases occur in foreign-born persons. Previous studies have shown that majority of active disease cases in immigrants/expatriates originating from TB endemic countries occur as a result of reactivation of previously acquired infection mostly within two years of their migration [8, 12, 171, 172]. Application of IGRAs to identify latently infected individuals and their treatment for LTBI has greatly helped in lowering the incidence of TB in rich, advanced countries [171, 172, 182, 186, 187]. Some other low-intermediate TB incidence countries which contain large expatriate populations originating from TB endemic countries [188–192] are also evolving similar strategies for controlling TB [12, 193].

## 14. Conclusions

With nearly 9 million new active TB cases and 2 million deaths occurring every year, TB remains a major infectious disease of global proportion. Active disease patients with sputum smear-positive pulmonary TB are the main source of infection. Primary infection with *M. tuberculosis* leads to clinical disease in ~10% of individuals. In the remaining cases, the ensuing immune response arrests further growth of *M. tuberculosis*. However, the pathogen is eradicated completely in ~10% people while the immune response in the remaining ~90% individuals only succeeds in containment of infection as some bacilli escape killing by blunting the microbicidal mechanisms of immune cells and remain in nonreplicating (dormant or latent) state in old lesions. The dormant bacilli retain their ability to induce reactivation and to cause active TB if a disruption of immune response occurs. While active transmission is a significant contributor of active disease cases in high TB burden countries, most cases in low TB incidence countries arise from this pool of latently infected individuals. The positive tuberculin skin test or more recent and specific T cell-based IGRAs in a person without overt signs of the disease indicates LTBI. Two commercial IGRAs, QFT-G-IT and T-Spot-TB, are also available. Application of IGRAs to identify latently infected individuals and their treatment for LTBI has greatly helped in lowering the incidence of TB in rich, advanced countries. Similar approaches also hold great promise for other countries with low-intermediate rates of TB incidence.

## Figures and Tables

**Table 1:** Some important *M. tuberculosis* factors that modulate the innate immune response and promote persistence of the pathogen leading to latent tuberculosis infection.

*M. tuberculosis* component	Immune cell process inhibited/affected	Reference(s)
19 kDa Lipoprotein (LpqH)	MHC class II expression and antigen presentation	[32, 44, 85–87]
19 kDa Lipoprotein (LpqH)	Phagosomal processing by MHC class I pathway	[88]
Mannose capped lipoarabinomannan	Phagolysosome biogenesis	[46, 89, 90]
Mannose capped lipoarabinomannan	MHC class II expression and antigen presentation	[85, 90]
Mannose capped lipoarabinomannan	IL-12 secretion of dentritic cells/macrophages	[45, 90]
Mannose capped lipoarabinomannan	Apoptosis of macrophages	[90, 91]
Trehalose dimycolate (cord factor)	Phagolysosome biogenesis	[92, 93]
Trehalose dimycolate (cord factor)	MHC class II expression and antigen presentation	[94]
6-kDa early secreted antigenic target (ESAT-6)	Pathogen containment in phagolysosome/macrophage	[95–97]
ESX-1 secreted proteins	Macrophage proinflammatory cytokine response	[98]
Serine/threonine protein kinase G (PknG)	Phagolysosome biogenesis	[99, 100]
Lipid phosphatase (SapM)	Phagolysosome biogenesis	[101]
Lipoprotein LprA	MHC class II expression and antigen presentation	[36]
Lipoprotein LprG	MHC class II expression and antigen presentation	[35]
Secretion system SecA2	Apoptosis of macrophages and dendritic cells	[102]
Superoxide dismutase (SodA)	Apoptosis of macrophages and dendritic cells	[102]
NADH dehydrogenase (NuoG)	Apoptosis of macrophages and dendritic cells	[103]
